# Breast Cancer among Cancer Patients Visiting the Department of Internal Medicine of a Tertiary Centre

**DOI:** 10.31729/jnma.8466

**Published:** 2024-02-29

**Authors:** Rakshya Shrestha, Bishal Paudel, Bishal Panthi, Bindu Gyawali, Anup Pandey, Surendra Khanal, Siddinath Gyawali

**Affiliations:** 1 Department of Internal Medicine, Tribhuvan University Teaching Hospital, Maharajgunj, Kathmandu, Nepal; 2 Curative Service Division, Department of Health Services, Ministry of Health and Population, Teku, Kathmandu, Nepal; 3 Piedmont Athens Regional Medical Center, 1200 Prince Avenue, Athens, Georgia, USA; 4 Maharajgunj Medical Campus, Maharajgunj, Kathmandu, Nepal

**Keywords:** *breast cancer*, *cross-sectional study*, *demography*, *malignancy*, *prevalence*

## Abstract

**Introduction:**

Breast cancer is one of the most common cancers worldwide both in terms of incidence and mortality. Its incidence has been on an increasing trend in developing nations including Nepal, however, there is very limited evidence of its demographic profile in our setting. This study aimed to find out the prevalence of breast cancer among cancer patients visiting the Department of Internal Medicine of a tertiary centre.

**Methods:**

A descriptive cross-sectional study was conducted among cancer patients visiting the Department of Internal Medicine of a tertiary care centre using retrospectively collected data from 1 August 2022 to 30 July 2023 after obtaining ethical approval from the Institutional Review Committee. Histopathologically confirmed cancer patients were included and those with incomplete and duplicated data were excluded. A convenience sampling method was used. The point estimate was calculated at a 95% Confidence Interval.

**Results:**

Among 2067 cancer patients, the prevalence of breast cancer was 102 (4.93%) (4.00-5.87, 95% Confidence Interval). The mean age was 50.51±2.08 years. The most commonly affected age group was 40 to 60 years constituting 61 (59.80%) patients. Histologically, invasive breast cancer of no special type was the most common and was found in 91 (89.22%) patients.

**Conclusions:**

The prevalence of breast cancer was similar to other studies done in similar settings.

## INTRODUCTION

Breast cancer is the most common cancer worldwide and occupies the first position in terms of incidence and fifth position in terms of mortality.^1,2^ In Nepal, breast cancer occupies the third position in the whole population and second position among females in terms of incidence with 1,973 new cases in 2020.

Similar to other developing nations, in the present scenario, Nepal is facing the overwhelming burden of non-communicable diseases (NCDs). Analysis has shown that the incidence of breast cancer increased by 108.57% from 1990 to 2017.^3^ Similarly, an analysis of 3270 patients in a cancer hospital in Nepal showed that 5.59% had breast cancer.^4^ Despite the increasing prevalence of breast cancer in Nepal, there are limited studies on its demographic and epidemiological aspects.

This study aimed to find out the prevalence of breast cancer among cancer patients visiting the Department of Internal Medicine of a tertiary centre.

## METHODS

This was a descriptive cross-sectional study conducted among cancer patients visiting the Department of Internal Medicine (Medical Oncology) of Tribhuwan University Teaching Hospital, Maharajgunj, Kathmandu, Nepal. Data from 1 August 2022 to 30 July 2023 were retrieved from the computer records system on 11 September 2023. Ethical approval was obtained from the Institutional Review Committee of the Institute of Medicine (Reference number: 239 (6-11) E2 080/081). Data were anonymised and deidentified and confidentiality was maintained. Cancer patients visiting outpatient departments during the study period with complete data were included. The patients with secondary breast malignancies, those who have not undergone breast tissue biopsy were excluded from the study. A convenience sampling method was used. The sample size was calculated by using the following formula:


$$\begin{array}{ll} \text{n} & ={\text{Z}}^{2}\times \frac{\text{p}\times \text{q}}{{\text{e}}^{\text{2}}}\\ & =1.96^{2}\times \frac{0.056\times 0.944}{0.01^{2}}\\ & =2028 \end{array}$$

Where,

n = minimum required sample size
Z = 1.96 at 95% Confidence Interval (CI)
p = prevalence taken from previous study, 5.59%^4^
q = 1-p = 0.94
e = margin of error, 1%

The calculated sample size was 2028. However, 2067 patients were included.

The data were collected using a predesigned proforma. The demographic factors of the patients included age and their caste. The diagnosis of breast cancer was done according to the histological classification of breast cancer by WHO.^5^

The data obtained were tabulated in Microsoft Excel 2016. The statistical analysis was done using IBM SPSS Statistics version 21.0. The point estimate was calculated at a 95% CI.

## RESULTS

Among 2067 cancer patients, the prevalence of breast cancer was 102 (4.93%) (4.00-5.87, 95% CI). Most of them 61 (59.89%) belonged to the age group 41-60 years and only 20 (19.61%) of them were less than 40 years old. The mean age of the patients is 50.51±12.08 years. The median, minimum, and maximum age of the patients were 49, 25, and 83 years respectively. Only 1 (0.98%) patient was male. The maximum proportion of the participants 71 (69.61%) belonged to the Janajati category of ethnic division (Table 1).

**Table 1:** Demographic characteristics of patients with breast cancer (n = 102).

Variables	n (%)
**Age group (years)**	
>61	21 (20.59)
<40	20 (19.61)
41-60	61 (59.80)
**Sex**	
Female	101 (99.02)
Male	1 (0.98)
**Caste**	
Janjati	71 (69.61)
Brahmin/Chetteri	22 (21.57)
Dalit	5 (4.90)
Others	4 (3.92)

Histopathologically, 91 (89.22%) hadbreast cancer of nospecial type (NST) and only 1 (0.98%) patient had intraductalpapilloma and metastatic breast cancer each (Table 2).

**Table 2:** Histological diagnosis of the patients with breast cancer (n = 102).

Diagnosis	n (%)
Invasive breast cancer of no special type	91 (89.22)
Invasive ductal breast carcinoma	6 (5.88)
Invasive mucinous carcinoma	3 (2.94)
Intraductal papilloma	1 (0.98)
Metastatic breast carcinoma	1 (0.98)

## DISCUSSION

The prevalence of breast cancer in our setting was 4.93%. The prevalence of breast cancer detected in our study corresponds with a study conducted in a similar setting in Nepal which showed a prevalence of 5.59%.^4^ However, the prevalence of the disease identified in this study is less than that of national statistics which was 9.6% in 2020.^2^ In addition, the finding also contradicts the global data where breast cancer occupies 11.7% of all malignant cases in the world.^6^

Initially known as the disease of developed nations, breast cancer is an increasing trend among low- and middle-income nations. The increasing trend in the incidence of breast cancer in these nations is ascribed to increased risk factors including early menarche, late menopause, nulliparity, late age at delivering the first child, usage of hormone replacement therapy, postmenopausal obesity, physical inactivity, smoking, insufficient vitamin supplementation, excessive intake of processed food, and alcohol intake.^7-9^ Similarly, death rates due to breast cancer in these nations also outnumber those compared to the developed world.^8^

Our study revealed that the mean age of detection of breast cancer is 50.51 years (standard deviation 12.08). The median, minimum, and maximum age of the patients are 49, 25, and 83 years respectively. This finding corresponds to a study in which the mean, median, minimum, and maximum age of the patients with breast cancer was 49, 48, 22, and 82 years respectively.^4^ In addition, other similar studies conducted yield similar results with mean age ranging from 45 to 52 years.^9-12^ These scenarios of age characteristics of patients with breast cancer in these studies are consistent with the fact that an age of more than 40 years is a risk factor for the development of breast cancer.^13^

Only 1 (0.98%) patient with breast cancer was male. This finding corresponds with the finding in other studies where less than 1% of the patients with breast cancer are male.^8,11,14^ The lower prevalence of breast cancer in males is due to the presence of insignificant estrogen levels in males. Disruption of circulating levels of sex hormones, particularly estrogen, progesterone, and androgens is associated with an increased risk of breast cancer in females.^8^

According to the latest national census of Nepal (2021), Chhetri (16.4%) and Brahmin (11.3%) comprise the maximum proportion in terms of caste-wise classification of the national population.^15^ However, in the present study, Janajati occupied 69.61% of total cases with breast cancer while in combination, 21. 57% of patients belonged to Brahmin and Chhetri ethnicities. A similar finding with a higher proportion of the Newar communities, a caste belonging to Janajati ethnicity, was demonstrated by a similar study conducted among patients with breast cancer in Kathmandu.^4^ The higher residence of the Newar community in central Nepal, including Kathmandu Valley might be the reason behind this finding in our study.^15^

This study showed that most of the patients (89.22%) had invasive breast cancer of no specific type (NST). This type of breast cancer is the one that is diagnosed by default and fails to fall under one of the histological types. It is the most common type of breast cancer with a prevalence range of 40% to 80%.^8,16^

The data were collected retrospectively, therefore all the aspects of the sociodemographic profile of the patients were not included. Similarly, the possible risk factors of breast cancer in our setting could not be studied. Being a singlecenter study conducted in urban Nepal it does not reflect the actual demographic profile of the patients with breast cancer in Nepal. Therefore, we suggest a multicentre study with a cohort study design to evaluate the actual sociodemographic profile and to establish the causality in patients with breast cancer in Nepal.

## CONCLUSIONS

The prevalence of breast cancer among cancer patients was similar to other studies done in similar settings. The cohort study with an evaluation of risk factors in our setting is recommended for future studies. Thre is a need for targeted interventions and awareness programs to address risk factors associated with breast cancer and promote early detection.
